# Supplementary material for: Validation of a tissue-based predictive RNA test for immunotherapy benefit in NSCLC: the PREDAPT study
Source: NPJ Precis Oncol. 2026 Jul 29;10:369. doi: 10.1038/s41698-026-01632-6 (PMC13638978; doi:10.1038/s41698-026-01632-6)
Supplement: Supplementary file 1 [file 41698_2026_1632_MOESM1_ESM.pdf]

Table S1: Excluded Patient Samples

None (included in study)	89
Unacceptable Treatment Regimen	5
Other Concurrent Cancer	2
Unacceptable Specimen Site	14
Incomplete Data	5
Insufficient Material	19
Not NSCLC diagnosis	1
Tumor cellularity	7
Fail RNA QC	27
Fail Library QC	0
Fail Analytical QC	7
Withdrawn	0
**Total**	**176**

Table S2. Performance metrics for OncoPrism-NSCLC and PD-L1 TPS

	**Accuracy**	**PPV**	**NPV**	**Sensitivity**	**Specificity**
**OncoPrism** (Low+Medium vs High)	66.3%	71.4%	63.9%	47.6%	83.0%
**PD-L1** (TPS<50 vs TPS≥50)	54.2%	57.1%	52.7%	38.1%	70.7%

*PPV=Positive Predictive Value; NPV=Negative Predictive Value

Table S3. Performance metrics for OncoPrism-NSCLC and PD-L1 TPS in monotherapy patients (n=23)

	**Accuracy**	**PPV**	**NPV**	**Sensitivity**	**Specificity**
**OncoPrism** (Low+Medium vs High)	73.9%	70.0%	76.9%	70.0%	76.9%
**PD-L1** (TPS<50 vs TPS≥50)	52.2%	47.1%	66.7%	80.0%	30.8%

*PPV=Positive Predictive Value; NPV=Negative Predictive Value

Table S4: Multivariate Cox Proportional Hazards Model for PFS

Variable	Level	HR	95% Confidence Intervals	p.value
OncoPrism-NSCLC	High vs. Medium + Low	0.394	0.201-0.772	0.007
Treatment	Chemo-immunotherapy vs. ICI monotherapy	0.622	0.323-1.197	0.155
PD-L1 TPS	TPS≥50 vs. TPS<50	0.84	0.439-1.606	0.598
Histology	Squamous vs. Adenocarcinoma	1.437	0.786-2.626	0.239
Histology	Other vs. Adenocarcinoma	0.176	0.024-1.302	0.089

Table S5: Multivariate Cox Proportional Hazards Model for OS

Variable	Level	HR	95% Confidence Intervals	p.value
OncoPrism-NSCLC	High vs. Medium + Low	0.407	0.204-0.81	0.011
Treatment	Chemo-immunotherapy vs. ICI monotherapy	0.633	0.323-1.243	0.185
PD-L1 TPS	TPS≥50 vs. TPS<50	1.021	0.516-2.019	0.952
Histology	Squamous vs. Adenocarcinoma	1.202	0.646-2.238	0.562
Histology	Other vs. Adenocarcinoma	0.262	0.035-1.944	0.19

Table S6. OncoPrism and PD-L1 predicted and actual outcomes

	PD-L1 TP	PD-L1 FN	PD-L1 TN	PD-L1 FP
OncoPrism TP	11	9	-	-
OncoPrism FN	5	17	-	-
OncoPrism TN	-	-	26	8
OncoPrism FP	-	-	3	4

*TP=True Positive; FN=False Negative; TN=True Negative; FP=False Positive

Figure S1

A

PFS

Subgroup	Level	n	HR	p
NSCLC	all	89	0.369	0.001
Sex	female	38	0.476	0.092
	male	51	0.309	0.01
Histology	adeno	58	0.543	0.08
	squamous	26	0	0.998
ECOG	0	19	0.87	0.863
	1	55	0.456	0.034
	unknown	10	0	0.999
Eligibility	combo	63	0.488	0.06
	mono	26	0.049	0.004
Overall Stage	IVA	43	0.411	0.032
	IVB	30	0.378	0.081
	other	12	0.356	0.344
PD-L1	<1	37	0.554	0.278
	1-49	18	0.936	0.917
	50+	28	0.234	0.006

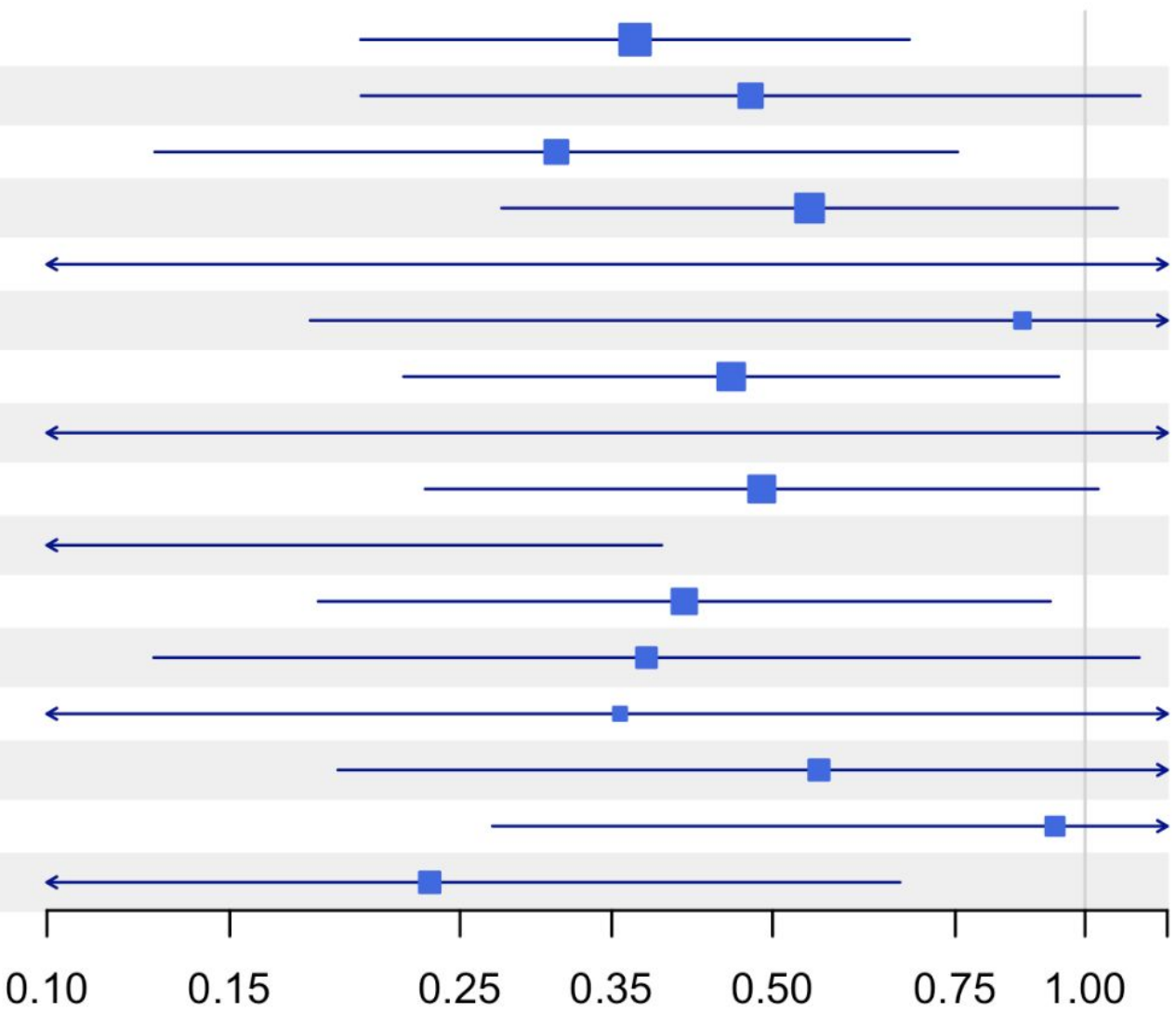

B

OS

Subgroup	Level	n	HR	p
NSCLC	all	89	0.389	0.004
Sex	female	38	0.327	0.032
	male	51	0.381	0.035
Histology	adeno	58	0.555	0.109
	squamous	26	0.094	0.025
ECOG	0	19	0.453	0.464
	1	55	0.369	0.013
	unknown	10	0	0.999
Eligibility	combo	63	0.515	0.099
	mono	26	0.101	0.003
Overall Stage	IVA	43	0.315	0.017
	IVB	30	0.463	0.177
	Other	12	0.283	0.259
PD-L1	TPS <1	37	0.47	0.225
	TPS 1-49	18	0.889	0.866
	TPS 50+	28	0.268	0.014

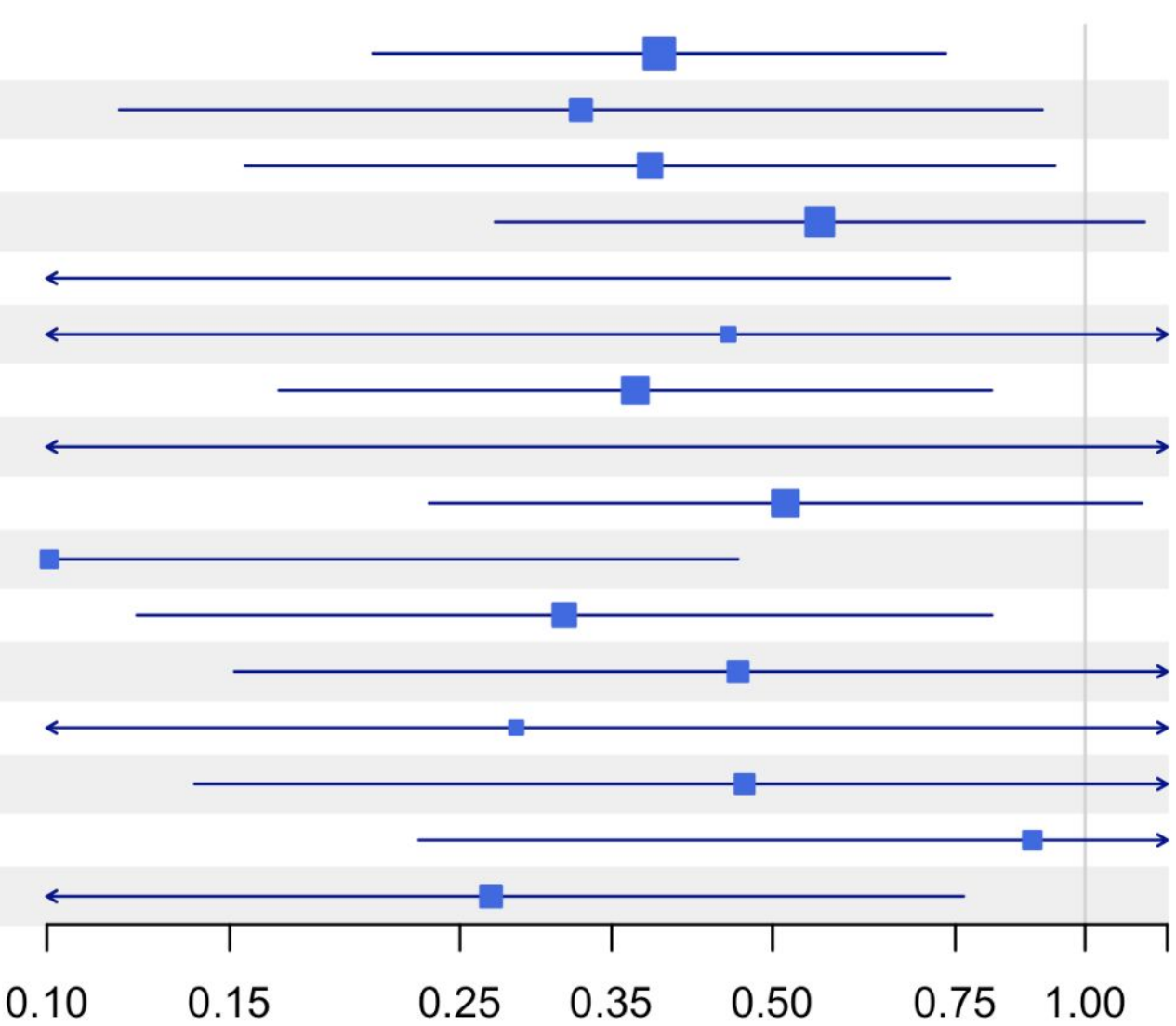

Figure S1. OncoPrism high group is associated with longer survival. Univariate Cox proportiational hazards modeling was used to interrogate the hazard ratios (HR) for high vs. low plus medium OncoPrism groups across the entire PREDAPT dataset as well as within potentially relevant clinical subgroups. **(A)** The progression-free survival (PFS) HR for OncoPrism high vs. medium plus low was significant across the entire cohort (n=89) as well as some subgroups. **(B)** The overall survival (OS) HR for OncoPrism high vs. medium plus low was significant across the entire cohort (n=89) as well as some subgroups.

Figure S2

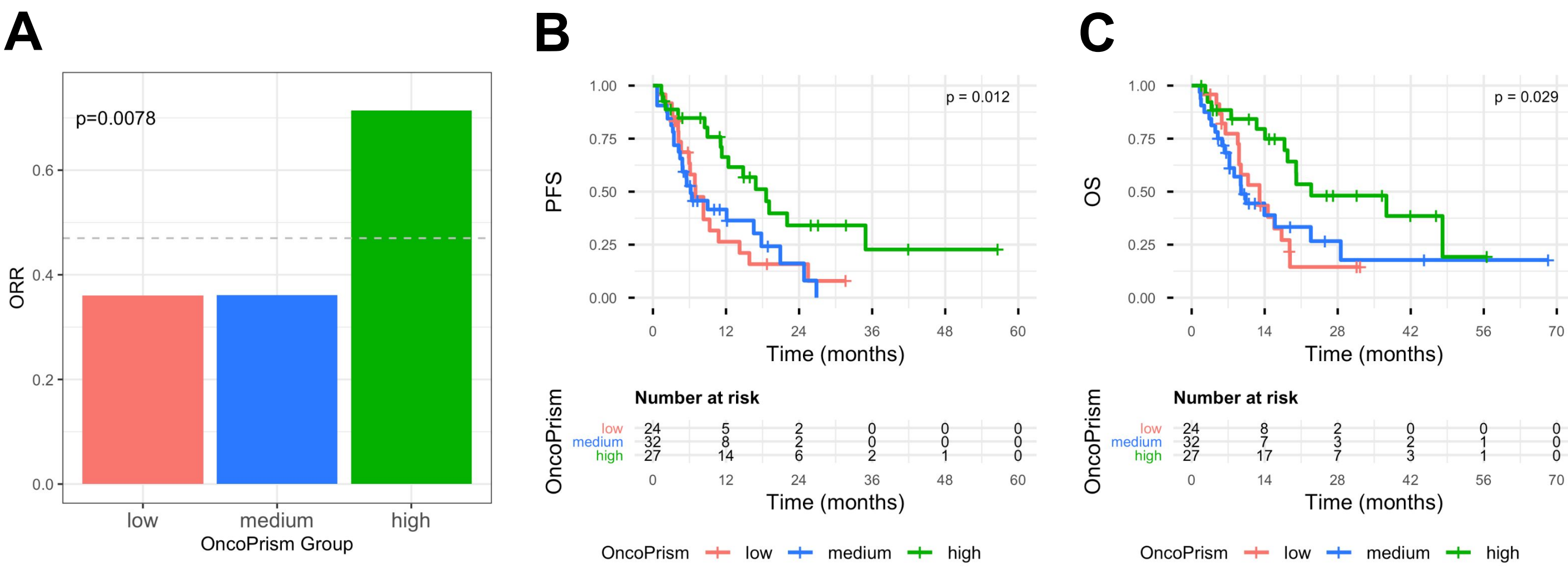

Figure S2. OncoPrism group is correlated with clinical benefit in the subset of PREDAPT patients where PD-L1 TPS data was available (n=83). (A) OncoPrism-NSCLC group was significantly correlated with overall response rate (ORR). The ORR for the entire cohort is shown as a dashed line. **(B)** OncoPrism-NSCLC significantly predicted progression-free survival (PFS). **(C)** OncoPrism-NSCLC significantly predicted overall survival (OS).

Figure S3

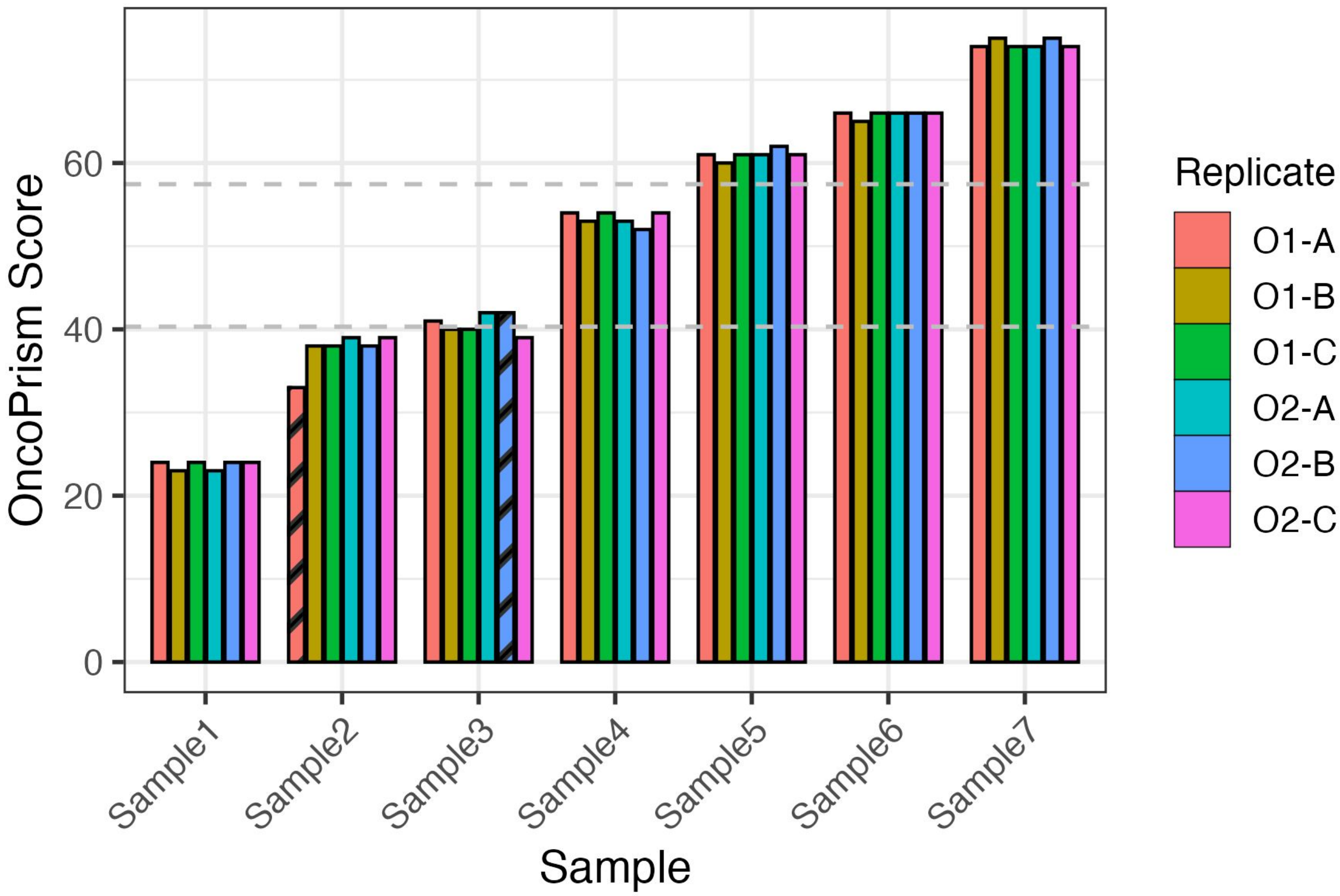

Figure S3. OncoPrism-NSCLC is analytically robust. Seven biological samples were processed in triplicate by two operators on different days with two different reagent lots for each batch. Striped boxes represent replicates that did not pass QC. OncoPrism group thresholds are shown as dashed lines. “O1” is Operator 1. “O2” is Operator 2.
